# The Metabolic Mind: Revisiting Glucose Metabolism and Justice Involvement in Neurolaw

**DOI:** 10.3390/neurosci6040120

**Published:** 2025-11-24

**Authors:** Alan C. Logan, Colleen M. Berryessa, Jeffrey M. Greeson, Pragya Mishra, Susan L. Prescott

**Affiliations:** 1 Nova Institute for Health, Baltimore, MD 21231, USA; susan.prescott@uwa.edu.au; 2 School of Criminal Justice, Rutgers University, Newark, NJ 07102, USA; colleen.berryessa@rutgers.edu; 3 College of Science and Mathematics, Rowan University, Glassboro, NJ 08028, USA; greeson@rowan.edu; 4 Department of Law, Allahabad University, Prayagraj 211002, India; pragya@allduniv.ac.in; 5 School of Medicine, University of Western Australia, Perth 6009, Australia; 6 School of Medicine, University of Maryland, Baltimore, MD 21201, USA

**Keywords:** metabolism, neurolaw, criminal justice, nutrition, mental health, diminished capacity, microbiome, aggression, neuropsychiatry, biological criminology

## Abstract

Neuropsychiatric interest in the relationship between glucose metabolism and criminal behavior dates back nearly a century. In particular, hypoglycemia was thought to play a causative role in some criminal acts, especially non-planned incidents involving impulsivity and in-the-moment risk-taking or aggression. While interest in carbohydrate metabolism in forensic populations faded in the 1990s, recent years have witnessed a renewed interest in metabolic dysfunction, mental health, and cognition. This area of research has grown increasingly robust, bolstered by mechanistic discoveries, epidemiological work, and intervention trials. Advances in microbiome (legalome) sciences, aided by omics technologies, have allowed researchers to match objective markers (i.e., from genomics, epigenomics, transcriptomics, and metabolomics) with facets of cognition and behavior, including aggression. These advances, especially the concentrated integration of microbiome and omics, have permitted novel approaches to the subject of glucose metabolism, and cast new light on older studies related to justice involvement. With current technologies and contemporary knowledge, there are numerous opportunities for revisiting the subject of glucose metabolism in the context of neurolaw. Here in this viewpoint article, we reflect on the historical research and emergent findings, providing ideation for future directions.

## 1. Introduction

In the mid-20th century, neuropsychiatrist Joseph Wilder argued that cognition and behavior otherwise associated with justice involvement—impulsivity, irritability, hostility, emotional reactivity, aggression, risk-taking, violence, and/or antisocial activities—could often be traced to dysregulated glucose control [1]. In particular, Wilder posited that hypoglycemia is a common observation among forensic populations [2,3]. Wilder was not alone in his contentions. In a 1966 report of 300 cases of hypoglycemia, 45% experienced irritability, 22% presented with antisocial behavior, and 10% reported suicidal tendencies [4].

In a 1943 case reported in the *Lancet*, experts linked a matricide defendant’s hypoglycemia with abnormal electroencephalogram (EEG) readings [5]. Based on expert testimony, the jury found the defendant temporarily insane (due to the hypoglycemia) at the precise time of the matricide. The jury decision meant 20-year-old Derek Thayer Lees-Smith was diverted to a medical institution rather than the gallows. The case received international media attention (with headlines such as ‘Blood Sugar Murder’) [6,7]. While rare, similar hypoglycemia-related acquittals have been noted in cases ranging from homicide to shoplifting [8,9,10,11,12,13,14]. In 1970s medical anthropology research, hypoglycemia was linked with higher rates of aggression in one area of South America [15]. These and other cases have been part of a decades-long controversy surrounding the role of sugar, hypoglycemia, and glucose metabolism, in relation to forensic neuropsychiatry [16].

This viewpoint article explores emergent discoveries in the area of glucose metabolism in neuropsychiatric conditions [17,18,19], arguing that metabolic differences may increase the risk of justice involvement. Emerging research, aided by advances in microbiome sciences and omics technologies, enables a re-examination of older findings related to carbohydrate metabolism in forensic populations. While some of the older findings might be considered anecdotal, a revisit opens new avenues of mechanistic understanding and offers new ideas for contemporary research designs.

Drawing from the PubMed, PsycINFO, Google Scholar, and Google Books databases, we revisit 20th century research through the lens of contemporary scientific research. We supplement the material found in academic databases with relevant articles drawn from media databases, including NewsBank and Ancestry’s Newspapers.com. As we outline below, mid-20th-century observations can be revisited with contemporary neurobiological tools, including neuroimaging, metabolomics, and continuous glucose monitoring. The empirical studies we highlight should therefore be read as illustrative rather than exhaustive, chosen to motivate a broader conceptual and agenda-setting argument.

**Figure 1:**
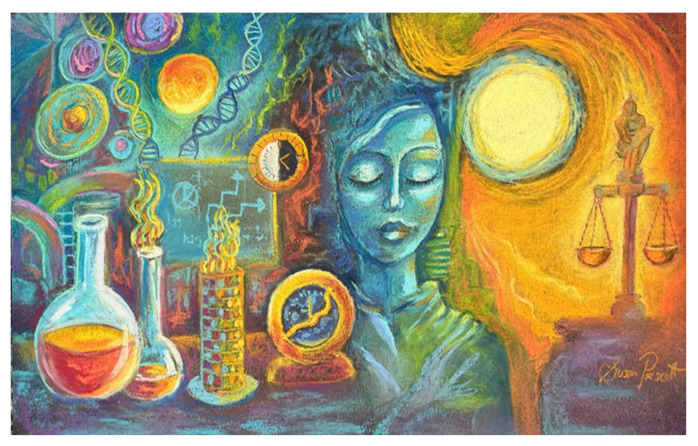
The Metabolic Mind and Justice: Emergent research is illuminating mechanistic pathways linking between metabolism and justice involvement (with permission of the artist, Susan L. Prescott, MD, Ph.D).

We contend that insights gained from glucose metabolism will support the much-needed ‘Copernican Revolution’ in 21st-century criminal justice—a transformed system that adapts to neuroscientific discoveries [20]. We suggest that metabolically informed science might contribute to more humane, proportionate, and rights-consistent responses to justice-related behaviors. We argue that future discoveries in this area will be of critical importance to neurolaw, the interdisciplinary field that aids in the translation of brain sciences into legal decisions and policy. Neurolaw challenges the prescientific structures of contemporary legal systems, rooted as they are in folk psychology ideas of moral blameworthiness and deserts-based punishments [21]. Beyond neurolaw per se, discussions of glucose metabolism and cognition/behavior are of relevance to workplace performance and the wellness of professionals working within the criminal justice system [22] (Figure 1).

## 2. Glucose Metabolism

The human brain is an energetically demanding organ, consuming roughly 20–25% of the body’s total glucose supply in adults, with the developing brain requiring even more. Around 30% of circulating glucose is present in the brain’s extracellular fluid, and stabilization of cerebral glucose typically occurs within thirty minutes of marked peripheral blood glucose changes [23]. Of the energy derived from glucose metabolism, approximately 70% is in direct support of neuronal signaling functions, including synaptic transmission, the propagation of action potentials, glutamate cycling, and postsynaptic calcium activities [24]. Glucose metabolism is also involved in the maintenance of resting membrane potential, axonal transport, cytoskeletal remodeling, and the manufacture of metabolites that influence inflammatory and redox pathways [25,26].

In classic neuroscience textbooks, various types of hypoglycemia have been identified. First, and arguably most common, postprandial (or reactive) hypoglycemia is characterized by systemic blood glucose levels that drop below 70 mg/dL or drop by 20 mg or more (vs. baseline glucose) in response to an oral glucose tolerance test (GGT). This rapid change in blood glucose is typically accompanied by an ‘adrenergic syndrome’ via the release of multiple counterregulatory hormones and physiological responses. The second type is a neuroglycopenic syndrome characterized by very low fasting systemic blood glucose levels, and the third type is characterized by normal or high levels of systemic blood glucose, accompanied by relative cerebral hypoglycemia [27]. The latter type is thought to result from reduced blood-to-brain transport of glucose [28,29,30]. These types are not mutually exclusive, and clinical manifestation may vary depending on environmental factors and psychosocial stressors [31,32].

## 3. Research in Forensic Populations

Notwithstanding the mid-20th-century anecdotes, scientific examination of hypoglycemia in forensic populations began in the 1980s. In a series of studies involving an oral GGT, researchers reported that Finnish offenders with antisocial personality disorder, intermittent explosive disorder, impulsive and habitually violent behavior, experience reactive hypoglycemia [33,34,35]. Antisocial personality disorder was characterized by a very high (initial) glucose response, decline into a hypoglycemia state, and a slow return to baseline glucose levels [36]. Intermittent explosive disorder was also associated with a low glucose nadir but a more rapid return to basal values [37]. Higher levels of insulin secretion were reported in young adults with antisocial personality and inmates with a history of violent offenses [38,39], and associations between systemic hypoglycemia and low levels of cerebral spinal fluid (CSF) 5-hydroxyindoleacetic acid (5-HIAA) were also observed [40]. Using an insulin clamp method, Finnish researchers found that low non-oxidative glucose metabolism was associated with higher recidivism rates among violent, antisocial offenders—explaining 27% of the variation in the recidivistic offending [41].

Although these studies have received little follow-up in carceral settings or research involving individuals involved with the justice system, more recent investigations in at-risk populations provide support. For example, suicide attempts by violent means [42] and expressed violence among suicide attempters [43] have been associated with higher CSF insulin levels. Among patients with first-episode drug-naïve schizophrenia, higher levels of fasting glucose and insulin are associated with suicidal behavior [44].

In general, rates of diabetes have been reported to be significantly higher among individuals involved with the justice system and/or within carceral settings [45], with particularly strong associations with violent crimes [46]. Given that mental disorders are far more common in carceral settings (essentially operating as de facto mental health institutions) [47], and many neuropsychiatric disorders have been linked to metabolic syndromes [48], the concentration in forensic populations should not be surprising.

## 4. Research in Non-Forensic Populations

In order to develop an understanding of how metabolic dysregulation might lead to justice involvement—distinct from the study of justice involved individuals—researchers can look to the general population. In a recent Japanese study involving 139 non-diabetic patients with medically unexplained symptoms (e.g., fatigue, dizziness, tension, palpitations, brain fog), hypoglycemia (defined as less than 70 mg/dL or 3.9 mmol/L) after an oral GTT was found in 92% of the subjects. Significant hypoglycemia (less than 50 mg/dL or 2.8 mmol/L) was found in 25% of the patients. Notably, autonomic symptoms were more frequent among those with rapidly elevated insulin levels in response to the GTT, even when blood glucose levels were within normal limits [49]. Similar studies involving subjects with medically unexplained postprandial symptoms have found hypoglycemia (after an oral GTT) in many (30–50%) of the participants [50,51].

In small studies involving otherwise healthy non-diabetic adults, the induction of hypoglycemia (or hyperinsulinemia resulting in lowered blood-to-brain glucose transfer) has been associated with a variety of neuropsychiatric changes. For example, in the period following an oral GTT, even mild hypoglycemia has been associated with higher scores on questionnaire measures of aggressiveness [52,53]. Symptoms have included feelings of unreality and hallucinations [54] and an overall feeling of tension [55]. It is possible that lowered blood glucose, whether in the periphery or centrally, is drawing out symptoms that reflect a baseline proclivity toward aggression [56]. On the other hand, experimental research has shown that anger associated with insulin-induced hypoglycemia is not predicated on measures of trait anger and anger suppression [57].

While two-to-six-hour oral GTT research has contributed much to the understanding of metabolism, cognition, and behavior, it has been widely recognized that an oral GTT is distinct from the everyday mixed meals consumed by humans. Other than paralleling the consumption of a large quantity of sugar-sweetened beverages without other foods, the oral GTT is not reflective of a typical meal. To overcome this, researchers have approached the subject of hypoglycemia from unique directions, exemplified by a 2014 study of 107 married couples. Participants recorded morning and evening glucose levels over 21 days, and each day during this period, they were asked to insert between 0 and 51 pins into a doll representing their spouse. The number of pins represented anger levels directed at the spouse. At the end of the study, couples engaged in a computer task where “winners” could administer aversive noises to their partner. Lower blood glucose predicted both more pins in the doll and more frequent and intense noise administration, linking glucose levels directly to aggressive impulses and behaviors [58].

## 5. Glucose Administration, Transport

If hypoglycemia can provoke cognition and behaviors that otherwise increase risks of justice involvement, it suggests that glucose administration, at least acutely, might have palliative value. Indeed, short-term elevations in blood glucose have been reported to reduce tension [59] and aggression [60]. Moreover, the acute consumption of glucose beverages attenuates self-control impairments among adult volunteers engaged in experimental tasks intended to place cognitive (and glucose energy) demands on self-control [61].

In 1960, neurologist Barry Wyke reported that a subset of neuropsychiatric patients with aggression experienced relative cerebral hypoglycemia (RCH) despite normal blood glucose levels in the periphery. He reported that RCH and its associated sudden-onset aggression could be relieved by raising blood glucose by 50% [62]. Absent currently available technology, Wyke could only speculate that RCH was a matter of inefficient or dysfunctional glucose delivery to nerve cells. In any case, Wyke was convinced that a small segment of the population, especially concentrated in people with neuropsychiatric disorders, experienced RCH. Wyke theorized that “*in these particular individuals the cerebral glucose requirement is higher, or the rate of passage of glucose into the brain is slower*” [63]. Of course, it could be both.

Today, we know that the GLUT family of transcellular transporters governs glucose delivery to the brain. These mediate over 95% of glucose entry into neural tissue and display regional variability within the brain. Among these transporters, GLUT3 plays a central role in cerebral glucose metabolism, supporting the excitatory–inhibitory balance of neural circuits, shaping neurodevelopment, and enabling activity-dependent plasticity [64]. GLUT3 is encoded by the SLC2A3 gene, and copy number variants of SLC2A3 have been linked to various neuropsychiatric disorders, including attention deficit hyperactivity disorder (ADHD) and bipolar disorder [65]. In preclinical models, GLUT3 knockout has been associated with anxiety, impulsivity, and aggression [66,67], while increased GLUT3 expression is associated with enhanced glucose uptake in the prefrontal cortex and mitigation of depressive-like behaviors [68].

Neuroscientists are currently making significant strides in understanding how neurons use glucose [69], and to what extent neurons and astrocytes operate as a metabolically coupled unit, simultaneously meeting the energetic demands of neurotransmission and providing neuroprotection [70]. Indeed, emergent research points toward insulin resistance and metabolic disturbances in neuropsychiatric conditions, leading to a cascade of neuroinflammation, oxidative stress, and impairments in brain mitochondrial function [71,72]. Individual differences in mitochondrial (dys)function may be a key factor in impulse control [73]. Given recent findings on insulin resistance, expression of glucose transporter proteins, and mitochondrial dysfunction in neuropsychiatric conditions, Wkye’s theory of relative cerebral hypoglycemia may have credibility. Further mechanistic scrutiny and evaluations of causal relationships await.

## 6. Acute Glucose vs. Dietary Patterns

Although research suggests that administering pure glucose may have value in curbing aggression and antisocial behavior, the continued administration of high amounts of sugar is not a viable solution. Excess dietary sugar intake over the long term has been associated with numerous non-communicable diseases, including depression and anxiety [74], and may promote the very behaviors under scrutiny [75]. For example, long-term intake of excess sugar, especially in early life, has been associated with higher risks of violence and justice involvement [76,77,78]. Prospective research indicates that the progression of metabolic glucose dysregulation is associated with increased impulsivity [79]. In preclinical models, diets high in processed fats and added sugar have been shown to drive impulsive choice behavior [80].

Relationships between unhealthy and healthy dietary patterns and neuropsychiatric disorders are complex, with evidence showing bidirectional causal pathways. For example, prospective research shows that externalizing behavior in pre-school [81] and parent-rated internalizing behavior in 8-year-old children [82] is associated with higher subsequent intake of dietary sugar. While impulsivity may drive unhealthy dietary choices, including the consumption of sugar-rich foods and beverages, it is also possible that the consumption of sugary foods serves as a form of self-medication in reducing distress in the short term [83,84]. Here, it is worth noting that ultra-processed food addiction is emerging as a legitimate clinical entity [85], and recent findings in a forensic population show that higher scores on the Eating Disorder Examination Questionnaire predict increased spontaneous aggression and excitability [86]. Oral GTT research shows that non-diabetic adults living with food addiction are twice as likely to experience reactive hypoglycemia compared to same-weight adults without food addiction [87]. Adverse childhood experiences, already well known to increase risks of subsequent justice involvement, are associated with higher odds of ultra-processed food addiction [88].

Moving beyond the oral GTT, researchers have examined cognitive and behavioral responses to test meals of varying macronutrient content. For example, the acute consumption of carbohydrate-rich (and protein-poor) food can stabilize mood and attenuate stress reactivity in stress-prone adults subjected to experimental stressors [89,90]. On the other hand, subjects consuming a high-carbohydrate breakfast (vs. a breakfast with a more balanced carbohydrate/protein/fat ratio) are more likely to engage in social punishment behavior in response to others’ norm violations [91]. In adolescents, skipping breakfast is associated with higher rates of impulsivity, impaired attentional control, and higher depressive symptoms [92]. At least one study has shown that lower glycemic index diets high in fruits, vegetables, legumes, eggs, and high-quality protein sources are associated with a decreased risk of aggression [93].

Studies using meals with differing macronutrient contents force discussions of temporal satiety and hunger. That is, high-carbohydrate meals (vs. mixed meals with protein and fat) are capable of causing rapid elevations in blood glucose and an early glucose nadir, the consequences of which appear to include a faster onset of hunger in the 3 to 4 h postprandial period [94] and greater brain activity in areas related to reward and craving (e.g., right nucleus accumbens) [95]. The size of the postprandial dip in blood glucose (relative to baseline) predicts more intense hunger 2 to 3 h later [96]. This matters because hunger, even when blood glucose levels have returned to baseline, is distinctly associated with elevated anger, irritability, tension, and confusion [97,98]. Indeed, chronic hunger, as experienced in at-risk persons and communities, may provide partial explanations for the links between food insecurity and crime [99]. In particular, food insecurity has been associated with aggression and violent crime [100,101]. People living with food insecurity are reliant upon inexpensive food high in sugar and devoid of fiber and are almost 4 times more likely to report clinically significant patterns of addictive-like ultra-processed food intake [102,103].

In sum, the available research suggests that the acute administration of glucose can curb short-term aggression and improve self-control in healthy adults in laboratory settings; on the other hand, a significant body of research indicates that long-term sugar intake, or extended post-prandial periods (e.g., 2 to 4 h) after a high-glycemic index meal, is associated with negative emotional states. While this might be viewed as a “sugar paradox” it presents a relatively straightforward path for detailed study. How might a metabolic inflection point differentiate benefit vs. risk over time? If repeated post-prandial spikes contribute to insulin resistance, low-grade inflammation, and gut dysbiosis, it is reasonable to suggest that downstream shifts remodel neural reward circuits and prefrontal control. This pathway reflects the contemporary carbohydrate-insulin model of obesity; here, high glycemic-index foods are thought to lead to rapid reductions in blood glucose in the late postprandial phase. In turn, the brain perceives the low levels of metabolic fuel to be a signal of “cellular semistarvation,” which further stimulates hunger and cravings for additional high-glycemic load foods—in an attempt to rapidly raise blood glucose [104]. Through various direct and indirect pathways, the gut microbiome, a topic we will explore next, can influence both blood glucose levels [105] and hunger [106].

## 7. Microbiome and Omics

Over the last two decades, the idea that gut microbiota can influence mood and cognition has evolved from a fringe concept [107,108] to one of the most exciting areas of research within neuropsychiatry. Aided by mechanistic understandings of gut microbe-brain connections, emergent research is demonstrating that gut microbiota may play a role in aggression, impulsivity, and/or antisocial behaviors [109]. Relationships between gut microbiota and behaviors associated with justice involvement have been described in detail elsewhere [110,111]. Among the potential mechanistic pathways, the role of the gut microbiome (the microorganisms and their theater of activity) in blood glucose control is central to the current thesis.

Gut microbes manufacture numerous bioactive chemicals, including the short-chain fatty acid butyrate, which is known to have blood glucose-regulating properties [112]. Gut microbes also convert primary bile acids into secondary forms, the latter of which are capable of promoting incretin GLP-1 secretion for blood glucose regulation [113]. Circulating bile acids are positively associated with insulin resistance [114]. Excess production of branched-chain amino acids (or limitations on their degradation) as a result of an abundance of select gut microbes has also been implicated in blood glucose dysregulation [115,116]. Moreover, emergent intervention research is also demonstrating that oral probiotics and other agents targeting the gut microbiome can aid in glycemic control in adults with type 2 diabetes [117,118].

**Figure 2:**
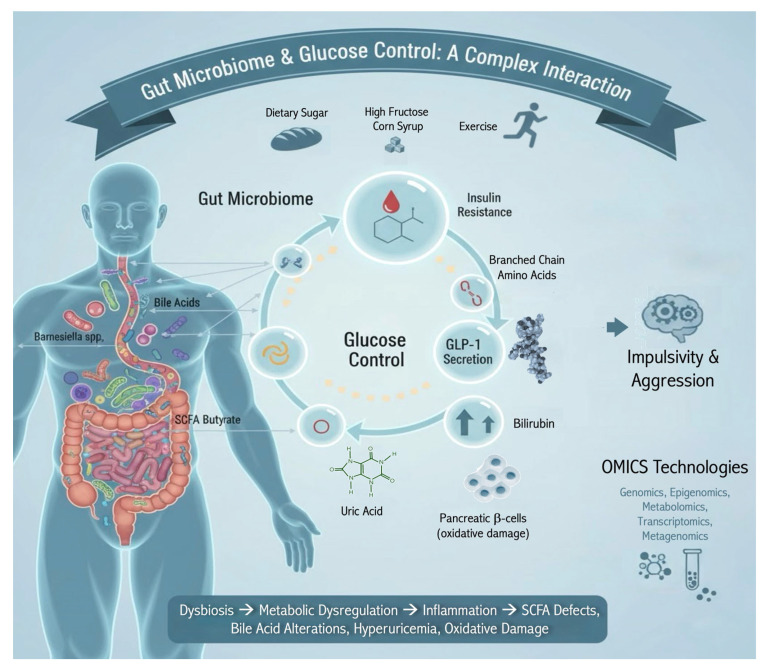
Dysbiotic Drift: Disturbances to the gut microbiome have been linked to metabolic dysregulation. Shifts in microbes may lead to low-grade inflammation, oxidative stress, and the production of various metabolic products with implications to justice-related behavior. Omics technologies and microbial signatures may provide key findings of value to prevention, treatment, and rehabilitation.

Given the far-reaching influence of gut microbes on metabolism, it is not surprising that specific gut microbial signatures are helping to explain the wide intra- and interindividual postprandial responses to glucose drinks or carbohydrate-rich test meals [119]. For example, when researchers add microbial features to a model that includes fasting glycemic measures and basic clinical features (e.g., serum cholesterol, body mass index, blood pressure), they can explain more than 60% of the inter-individual variance of postprandial plasma glucose concentrations [120]. In a study investigating the metabolic response to a standardized serving of bread, baseline microbiome features (relative abundance of microbial species and genes) emerged as highly predictive of individual postprandial glucose responses [121] (Figure 2).

Given the complexity of the gut microbiome, it is unlikely that one microbial species will help explain metabolic dysregulation, although there have been preclinical indications that the administration of *Barnesiella* spp. can improve glucose control [122,123]. This is interesting because low levels of *Barnesiella* spp. have been linked to schizophrenia [124], type 2 diabetes with sleep disorder [125], and in preclinical models of substance use disorder [126]. The role of *Barnesiella* spp. in maintaining a healthy intestinal barrier and limiting chronic low-grade inflammation has also been described [127].

It is also worth mentioning that dietary sugar and gut microbiome interactions may influence the risk of justice involvement through underappreciated mechanisms. For example, dietary sugars (especially in the form of high fructose corn syrup) are known to elevate uric acid, the purine breakdown product that has been linked to impulsivity and aggression [128]. Gut microbes are emerging as a significant mediator of systemic uric acid [129]. Bilirubin levels are also influenced by gut microbiota, and it is noteworthy that low bilirubin levels are characteristic of intermittent explosive disorder [130] and glucose dysregulation [131].

Separate considerations involve gut microbes as regulators of hormone production, including androgens [132]. The most dramatic cases of criminal behavior in association with anabolic steroids involve supraphysiological doses [133], and research has linked supplemental anabolic steroid use with glucose dysregulation [134,135]. However, elevated testosterone (unrelated to supplemental steroid use) continues to be associated with aggression and antisocial behavior [136,137], and potential mechanistic links between gut microbes and glucose metabolism warrant scrutiny.

Related to advances in gut microbiome science is the emergence of ‘omics’ technologies, including those that allow for the matching of objective markers (i.e., from genomics, epigenomics, transcriptomics, and metabolomics) with facets of cognition and behavior [138,139]. With a multi-omics approach in large cohorts, researchers can pull unprecedented amounts of biomolecular information from fecal samples, as well as blood, exhaled breath, and saliva. For example, various metabolites and epigenetic markers can be paired with polygenic risk scores to provide a more accurate composite of aggression risk [140]. Moreover, multi-omics approaches can help move scientific knowledge and discussions from correlation towards causation [141]. Specific to our discussion of glucose metabolism, an oral GTT does more than move blood glucose dynamics—it produces significant changes in various circulating metabolites [142,143].

In sum, dysbiosis appears to promote metabolic dysregulation via a number of mechanisms, including systemic low-grade inflammation, SCFA deficits, alterations in bile acid metabolism, bilirubin production, hyperuricemia, and oxidative damage to pancreatic β-cells (Figure 2) [144]. At the same time, many of the links between the gut microbiome and health conditions, including metabolic dysregulation, remain correlative. Moreover, most of the probiotic (and other agents targeting the microbiome) and brain-related and/or metabolic intervention studies remain small in scale [145]. As discussed below, researchers now have the capacity to improve on this and examine metabolomics in detail, including those related to physiological and behavioral responses to glucose. If, as we suspect, glucose dysregulation is at play in some aspects of aggression, violence, impulsivity, and antisocial behavior, there are multiple opportunities to mechanistically link rapidly evolving gut–brain-behavior research to metabolic endpoints.

## 8. Future Directions

By the 1990s, prior mid-to-late 20th century interest in glucose metabolism within at-risk and justice-involved populations had faded. The reasons for this are not entirely clear, but conflicting findings and criticism of poor methodology may have contributed to this fade [146]. Based on the emergent research linking diet, microbiome, and glucose regulation to mental health more broadly, there is a need to revisit the topic within the frame of criminal justice.

While much has been written on metabolic syndrome in relation to mental health (the so-called Metabolic Syndrome Type II [147]), very little of the contemporary discourse is directed at implications within the criminal justice system. To demonstrate the practical value of glucose metabolism in prevention, treatment, rehabilitation, and risk assessments [148], the research will need to be approached in new ways. In particular, a criminal justice frame will require better integration and translation of the siloed mechanistic bench science, epidemiological findings, and intervention studies. We suggest paying attention to the following areas, focused around four thematic clusters:

I. Mechanistic & Basic Science Research
  1. Expansion of preclinical mechanistic research, including efforts that help to identify potential causal links between carbohydrate metabolism, specific biological markers, and justice-related behavior.
  2. In preclinical models, identify microbial signatures might help to explain postprandial glucose responses and divergences in cognition and behavior; expand preclinical fecal transplant studies (with objective metabolic, immune, and neurochemical markers) to include human donors with impulse control disorders, aggression, anger attacks, or intermittent explosive disorder [109].
  3. Scrutinize glucose transporter expression, cerebral glucose use, and possible links to aggression, impulsivity, and other behaviors.
  4. Examine the role of glucose metabolism in mitochondrial dysfunction and behavioral changes.
  5. Investigate the differences between acute vs. chronic glucose administration and high/low glycemic index dietary patterns on behavior and reward circuits and prefrontal control.
II. Clinical and Intervention Studies
  1. Intervention studies should be conducted that include dietary changes, markers of glucose metabolism, and cognitive–behavioral outcomes. Research shows that targeted interventions can be effective in reducing dietary sugar intake [149], although research in this area is limited for justice-involved populations [150].
  2. Research designs should incorporate continuous glucose monitoring (CGM), especially in projects intended to explore relationships between diet, cognition, and behavior [48]. This includes designs with a laboratory stressor or real-world outcomes. Indeed, CGM could be used to study some of the controversies surrounding the so-called “hungry judge effect”—hunger and blood glucose as predictors of harsh judgements by decision-makers operating in the criminal justice system [22]. CGM can also be used to examine mindfulness and contemplative practices as possible interventions for glucose regulation [151].
  3. In human populations, identifying microbial signatures might help to explain postprandial glucose responses and divergences in cognition and behavior. Recent studies have identified differences in gut microbiota in adults in prison confinement [152,153], and much of the early work on glucose metabolism and criminal behavior involved adults in carceral settings. However, insofar as diet, social exclusion, and the isolating conditions of confinement influence the gut microbiome [154] and blood glucose [155], causal connections are difficult to tease apart.
  4. The topic of glucose metabolism and forensic neuropsychiatry should be considered through an exposome lens. Advances in exposome science allow for scrutiny of biological responses to total lived experiences (i.e., life course exposures, both positive and negative) as they interact with genes over time [156]. Exposome science can help identify how psychosocial factors—ranging from marginalization and toxic/pollutant exposures to adverse childhood experiences and food deserts—can explain both glucose dysregulation [157] and criminality [158].
  5. Glucose metabolism, impulsivity, and risk-taking should be evaluated in healthy and at-risk populations [159,160]. How might glucose metabolism differ between people engaged in so-called white-collar crimes (which have been linked to genetics [161,162] and higher risks of recidivism [163]) and other forms of crime?
  6. The role of glucose metabolism should be scrutinized as a possible (partial) explanation for the observed links between obesity and aggressive behavior, conduct disorder, antisocial personality disorder, and impulsivity [164,165,166]. Recent prospective cohort research demonstrating that early life obesity is linked with higher rates of lifetime criminal behavior (independent of race) requires further study [167].
  7. Laboratory research designs, intended to provoke social stress and cognitive demands, could combine continuous glucose monitoring with challenge meals (or oral GTT). Research using fMRI shows that in healthy adults engaged in cognitively demanding tasks, the lab induction of hypoglycemia is associated with impaired cognitive function and task-specific localized reductions in brain activation. As cognitive load increases under hypoglycemic conditions, neuroimaging indicates that there is increased activity in planning areas and recruitment of brain regions in an effort to limit dysfunction [168]. Given the findings of previous neuroimaging studies in adults with histories of aggression and violence—e.g., reduced activity in prefrontal structures and overactivity in the limbic system [169,170]—there is a need to better understand how hypoglycemia (or other metabolic changes) might influence both neuroimaging and outcomes such as impulsivity and aggression in at-risk populations.
III. Technological and Omics Applications
  1. Research must establish causal links between microbe-manufactured chemicals, glucose regulation, and cognition and behavior. In addition to the previously mentioned examples of uric acid and bilirubin, the gut microbe-produced chemical propionic acid has been linked to metabolic disruptions [171] and behavioral disturbances [172,173]. Research shows that in adults with disorders of gut–brain interaction (DGBI), oral glucose beverages (75 g) induce a range of symptoms associated with carbohydrate intolerance [174]. Given overlaps between DGBI and glucose dysregulation [175] and mental disorders [176], this is an area ripe for research.
  2. Multi-omics approaches should be incorporated into future research. Metabolomics and other omics technologies can provide explanatory power to previous observations. For example, Finnish researchers found that the combination of high insulin resistance, low insulin sensitivity, and high beta cell activity indices in adults with antisocial personality disorder appears to be mediated by the serotonin 2B (5-HT2B) receptor (carriers of a common 5-HT2B receptor gene mutation appeared protected) [177]. Contemporary multi-omics analyses can provide a more detailed understanding of the links between genetics, energy metabolism, and neuropsychiatric risk [178].
  3. Future studies should study the extent to which (and the mechanisms by which) psychotropic medications could curb risks of justice involvement via glycemic control [179]. For example, emergent research has found that adherence to ADHD medications is associated with diminished risk of subsequent criminality [180,181]. Given that ADHD is associated with poor glycemic control [182], and medications may have a mild hyperglycemic effect [183] and increase cerebral glucose uptake [184], this is an area worthy of scrutiny.
  4. The potential value of the revolutionary glucagon-like peptide 1 receptor (GLP-1) agonist drugs in at-risk and forensic populations should be assessed. Emergent research suggests that the GLP-1 class may have a benefit in reducing impulsivity [185]. Although these agents have a relatively low risk of inducing hypoglycemia [186], it is critical to examine whether baseline glucose metabolism could help determine who might benefit. In a recent case involving genetically driven obesity (Smith–Magenis syndrome), clinicians reported that associated aggression and violence were attenuated by the introduction of an injectable GLP-1 agent, worsened with a switch to poorly absorbed oral form, and reduced again with the reintroduction of the subcutaneous GLP-1 agonist [187].
  5. Neuroimaging and electroencephalogram testing, in concert with omics, microbial signatures, and interventions targeting healthy glucose metabolism should be incorporated into future work. For example, neuroimaging studies have shown that unhealthy dietary patterns are associated with smaller hippocampal and brain volumes [188,189], whereas healthy dietary patterns are associated with increased cortical thickness [190] and enhanced functional connectivity in the frontoparietal and temporo-occipital regions [191]. Remarkably, even a short-term (four-day) diet rich in added sugars and saturated fat compromises hippocampal-dependent learning and memory in adults [192]. While single ultra-processed and fast-food meals can lead to changes in post-prandial physiology [193,194] and cognition [195], there is a need to examine the role of glucose metabolism as part of any observed differences in neuroimaging. Moreover, there is a need to tease apart the place of carbohydrates and added sugars in discussions of ultra-processed foods. It could be that levels of processing are far less important than added sugar [196].
  6. The potential value of glucose metabolism and metabolomic markers in addition to and alongside certain forensic risk assessments should be considered. In one Finnish study, basal insulin levels were equivalent to or better than the commonly used Psychopathy Checklist Revised (PCL-R) as a predictor of recidivism in adults with a history of violent crime and alcohol use disorder [148]. Currently, even when setting aside the publication bias and poor-quality research behind common criminogenic risk assessments, the overall accuracy of many commonly used risk assessment instruments used in parole, probation, and sentencing is often in the range of poor to fair [197]. We suggest that biological markers could provide additional data about risk when combined with the results of common risk assessments.
IV. Legal, Ethical, and Policy Implications
  1. With appropriate informed consent and ethical guardrails in place [198], the greater inclusion of at-risk and justice-involved individuals, including those in carceral settings, in metabolic research is needed.
  2. A ‘justice lens’ should be incorporated into neuroscience and metabolism research. The topic of prison systems, and the criminal justice system writ large, often sits at the periphery of neuropsychiatric discourse. Yet, prison systems are being used as poorly prepared alternatives to mental health institutions [47]. For example, compared to military veterans without posttraumatic stress disorder (PTSD), veterans with PTSD are 59% more likely to be arrested for a violent offense, and 61% more likely to be justice-involved [199]. Given links between PTSD and glucose dysregulation [200], a justice lens can help to underscore the importance of transdisciplinary research.
  3. Future work should consider the implications of glucose metabolism research to professionals and decision-makers working within the criminal justice system. For example, burnout is a significant issue among law enforcement officers, lawyers, and judges [22]. Burnout has been linked with poor occupational performance, and among police officers, use of excessive force [201]. At the same time, burnout has been associated with insulin resistance [202], and adults with severe burnout symptoms exhibit significantly elevated levels of both glucose and insulin in response to an oral GTT [203].
  4. Future research should engage diverse populations with cross-jurisdictional data integration. Synchronize metabolomics, behavioral, and legal outcome datasets across countries and legal systems to understand how cultural, dietary, environmental, and policy differences modulate metabolism and behavior relationships.
  5. Education and scientific literacy should be enhanced moving forward. When used as a defense or mitigation strategy, findings from biological criminology and legal biopsychology can lead to heightened perceptions and assumptions surrounding a person’s future dangerousness [204,205]. The erroneous idea that biological aspects of criminal behavior are permanent, or that genetic influences equate to individual destiny, is enduring. The solution to this is not to ignore or suppress scientific realities (such as glucose dysregulation) or to label biological criminology as inherently wrong. Research shows that potential jurors with higher levels of scientific knowledge are less likely to support harsh sentencing in vignettes of violent crime [206]. This suggests that, among many factors, the problem of mass incarceration (and its associated problems) may be facilitated, at least partially, by deficits in scientific literacy [207].
  6. We must anticipate the ethical and legal implications of rapidly growing scientific research that demonstrates the degree to which biology influences justice involvement [208,209]. It is important to acknowledge that pseudoscientific biological theories (e.g., Cesear Lombroso and others in the late 19th through mid-20th centuries) have resulted in a variety of social harms, especially those related to eugenics [210]. However, with appropriate 21st century ethical approaches, evidence-based biological considerations can be incorporated into neurolaw and frameworks of non-retributive fairness [211].
  7. The emerging research connecting neuromicrobiology and omics with justice-related behaviors (i.e., the legalome) is already posing difficult questions for the courts, including matters of legal responsibility and punishment [212]. If the existing research on metabolic dysfunction and behavior is replicated and expanded upon, it will add strength to the field of neurolaw [213]. Up to now, courts have largely kept neurobehavioral sciences at arm’s length; however, as the evidence mounts, this stance may be increasingly viewed as anti-science and untenable [158]. Institutional and social responses to robust neuroscientific evidence will require new frameworks, deeper collaborations, and cultural shifts in how responsibility, punishment, and public safety are considered. A neurolaw of metabolism that stops at individual responsibility would be incomplete; if states and institutions help create obesogenic, hyper-processed food environments and then punish the behavioral sequelae of those environments, questions of structural responsibility and distributive fairness inevitably arise.

## 9. Conclusions

“*We are probably standing here at a beginning of a new scientific approach to the problem of crime. Many and careful investigations will be necessary in order to establish the proper place of this problem* [glucose metabolism] *within the framework of criminology and correctional medicine*” Joseph Wilder, MD, Forensic Neuropsychiatrist, 1947 [1].

Mid-20th-century clinicians, including Joseph Wilder, began linking disordered glucose metabolism with impulsivity, aggression, and other behaviors often leading to justice involvement. Wilder proposed that, at least in some cases, such traits reflected dysregulated glucose control rather than moral failure, noting hypoglycemia as common among forensic populations. Indeed, several well-publicized criminal cases resulted in acquittals or reduction in charges based on defense evidence of hypoglycemia. However, the subject of carbohydrate metabolism in relation to criminal behavior continued to be a matter of debate through the 20th century.

Recent years have witnessed renewed interest in the relationship between metabolic dysfunction and neurocognition and behavior. However, despite an increasingly robust body of evidence, the area of glucose regulation and risk of justice involvement has received little attention in the modern era. We suggest that the emerging evidence sheds new light on older forensic research, particularly with the rapid advances in microbiome and omics technologies. We propose that metabolic variation may influence behavioral regulation and justice outcomes, offering new pathways for a neuroscience-informed transformation of the criminal justice system—a long-awaited Copernican Revolution.

## Data Availability

No new data were created or analyzed in this study. Data sharing is not applicable to this article.
